# Peri-Implant Architecture Management With Customized Healing Abutment in Flowable Composite in Immediate Implants: Two Case Reports and Narrative Review

**DOI:** 10.1155/crid/5538611

**Published:** 2025-05-29

**Authors:** Jorge Andrés Velazco Dávila, Luis Fernando Rosales García, Laura Valeria Torres Agudelo, Gloria Cristina Moreno Abello

**Affiliations:** ^1^Department of Pathology and Oral Surgery, Faculty of Dentistry, Pontifical Xavierian University, Bogotá, Colombia; ^2^Department of Oral Rehabilitation, Faculty of Dentistry, Pontifical Xavierian University, Bogotá, Colombia; ^3^Department of Microbiology, Faculty of Dentistry, Pontifical Xavierian University, Bogotá, Colombia

**Keywords:** custom healing abutments, dental implant, emergency profile, immediate implants, PEEK

## Abstract

**Background:** The management of peri-implant tissue seeks to meet the aesthetic expectations of patients, with a smaller number of procedures that favor the development and maintenance of healthy peri-implant soft tissue. The customized healing abutment technique on immediate implants has demonstrated numerous functions for the success of restorations on implants.

**Objectives:** The objective of the study is to analyze the biological aspects of the customized healing abutment using PEEK and flowable composite to condition the peri-implant tissues by presenting a clinical case of a patient from the oral surgery service of Pontificia Xavierian University.

**Case Report:** A patient underwent the placement of two postextraction implants and customized healing abutments in the posteroinferior area.

**Discussion:** This technique is aimed at maintaining the volume of keratinized mucosa and creating an emergence profile for the future prosthesis and reducing the risk of peri-implantitis. The development of healthy peri-implant soft tissues is essential to achieve the aesthetics and biological success of implant-supported restorations in all stages of healing and tissue maturation, and the abutment is essential because it allows a biological seal that protects the bone tissue.

**Conclusion:** It was demonstrated that the use of the customized healing abutment with PEEK and flowable composite immediately after implant placement after dental extraction keeps the volume of the soft and hard tissues around the implants at the time of definitive rehabilitation.

## 1. Introduction

The management of peri-implant tissue seeks to meet the aesthetic expectations of patients and processes with a smaller number of procedures that favor the development and maintenance of healthy peri-implant soft tissue. Traditional processes involve several procedures, longer time, higher oral trauma, and greater discomfort for patients [1]. The greater the number of procedures, the greater the risk of complications. To reduce both the time and complications with the healing of soft and hard tissues around immediate implants, the customized healing abutment technique on immediate implants was created. It has been shown to reduce the risk of peri-implantitis and provides tissue maintenance. Soft areas around the implant and the volume of the keratinized mucosa are important for future implant prosthesis [2]. Bone loss after implant placement depends on factors like implant connection type, abutment used, bone atrophy, and implant orientation. After 2–5 months of healing, implants anchor to the bone through osseointegration. Early bone loss occurs after abutment connection and prosthesis loading. Factors affecting bone resorption include occlusal trauma, gingival biotype, implant torque, bone thickness, bacterial colonization, and implant position. To reduce these issues, “one-piece” implants were introduced to eliminate contamination and micromovement. Studies show that bone-level implants have less marginal bone loss, though differences between implant types were not significant after 1–5 years [3].

Several studies showed that a significant reduction in the volume of the alveolar process occurred between 6 months and 1 year after tooth extraction. They reported a 50% loss of the alveolar width, at least, with a smaller reduction in the alveolar height. Bone loss is particularly prominent in the first few weeks of healing due to the presence of granulation tissue and the contractile activity of myofibroblasts [4].

Immediate provisionalization significantly reduced volume loss and area shrinkage at the external layer when data were compared to a delayed rehabilitation strategy [4].

Covani et al. demonstrated the importance of preserving the position of hard and soft tissues after single-tooth extraction by means of a simple collagen plug filling the empty socket. The collagen plug, within an intact alveolus, might be sufficient to prevent extensive tridimensional collapse of the alveolar bone, thus favoring future rehabilitation [5].

Cosola et al. showed that using just collagen could be sufficient to induce proper new bone formation in particular clinical situations [6].

The process of marginal bone loss could slowly continue throughout life because of the lack of dental vascularization and also because the function of the periodontal ligament is missing [7].

Subsequent bone loss may also depend on the type of extraction and the disinfection of the socket. Aiuto et al. report that decontamination with the Er.Cr YSGG laser can help reduce the risk of peri-implantitis and complications. The photoacoustic effect exerted by this type of laser has been proven to be effective against many pathogens [8].

Crespi demonstrated [9] that the magnetoelectric extraction approach should be taken into account whenever appropriate because the incidence of postoperative adverse effects is lower than that of the conventional tooth extraction technique.

Therefore, some professionals have suggested this technique to provide a better emergence profile of the peri-implant tissues. The technique was described approximately 8 years ago by authors such as Akin in 2016 [10], who called it “The Anatomic Harmony Abutment.” Its purpose was to preserve the anatomical emergence shape with a sutureless sealing of the implant site to improve the predictability of manufacturing and delivery of the final restoration [10].

On the other hand, Finelle in 2017 described [11] the technique as “Sealing Socket Abutment.” The SSA protocol aims to “seal” the socket without the use of invasive techniques such as flaps, incisions, and sutures. The Akin and Finelle techniques use prefabricated CAD/CAM abutments [11]. As discussed elsewhere, [12], [13], and [14] described the technique using composite and flowable composite, to make the technique more accessible because the abutment is made after the placement of the dental implant with a material commonly used in the office that significantly reduces costs.

Customized healing abutments on dental implants are attachments manufactured in the mouth or previously prefabricated to connect to the implant platform at the time of placement and can be created in different materials, PEEK, titanium, composites, ceramics, acrylics, and PMMA, which are screwed to reproduce a suitable emergence profile for the implant-supported crown [1].

Among the previous options, PEEK is a tooth-colored synthetic thermoplastic polymer that belongs to the polyaryletherketone (PAEK) group that presents physical, mechanical, and biological properties for biomedical applications, which allow complete adaptation to the soft tissues in the mouth. It is used in various components in implant dentistry including implant fixtures, implant abutments, provisional abutments, and healing abutments; other applications include endocrowns and occlusal splints [1, 13].

Composite is one of the most used dental materials for direct restorations. It typically comprises a mixture of dental resins and some inorganic fillers. Composites contain two or more monomers to achieve the desired mechanical properties [1, 13].

For the manufacture of customized healing abutments on implants, two types of composite have been used: composites and flowable composites, on provisional PEEK or titanium abutments, to capture the contour of the postextraction socket in immediate implants [13].

Composites contain silica, ceramics, metals, and polymers in varying concentrations to enhance properties. Resin composite bonds with other materials to provide a precise 3D representation of peri-implant tissue, aiding laboratory transfer. It has low elasticity but high fracture resistance. Both ceramic and resin abutments show similar failure rates in tests, suggesting that resin composite could be used for healing abutments. However, some resin components are cytotoxic, so proper polymerization and polishing are necessary to prevent bacterial adhesion and free radical release [1].

This technique using flowable composite will make it easier to increase its use in the future as it does not require CAD/CAM technology, is cheaper, and can be easily prepared in the office [14].

Customized healing abutments have had results as predictable as postextraction implants that were sealed with bone substitutes and collagen membranes [14].

The use of customized healing abutments seems to be an appropriate option to create, support, and maintain both critical and subcritical contours of the tissues after an extraction and immediate placement of the implant in both aesthetic and posterior zones of the maxilla or mandible. This approach could positively influence peri-implant health, simplifying the whole treatment [12–14].

The aim of this article is to analyze the biological aspects of the customized healing abutment using PEEK and flowable composite to condition the peri-implant tissues by presenting two clinical cases of a patient from the Oral Surgery service of the Pontificia Universidad Javeriana of the city of Bogotá, Colombia.

## 2. Case Report

**Figure 1:**
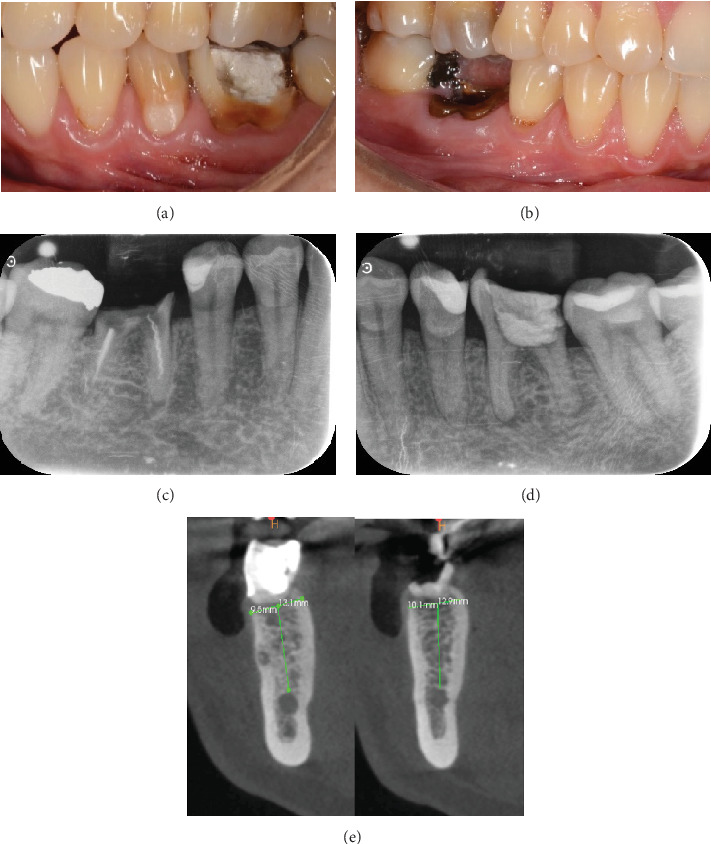
Preoperative situation: (a) clinical situation of Tooth 19, (b) clinical situation of Tooth 30, (c) initial periapical x-ray of Tooth 30, (d) initial periapical x-ray of Tooth 19, and (e) CBCT area of Teeth 19 and 30, respectively.

A 60-year-old woman who was referred to the Osseointegration Service of the Postgraduate Program in Pathology and Oral Surgery of the Pontificia Universidad Javeriana due to a coronal and root fracture in Tooth 30 and a nonrestorable root rest at the level of Tooth 19. Clinically, a complicated crown–root fracture was observed at the level of Tooth 30 and root rest at the level of Tooth 19 (Figure 1a,b).

Radiographically, a coronal radiopaque area is observed in the pulp chamber compatible with temporal cement, lumens of the mesial and distal root canals are observed, and a periapical radiolucent area is observed in the distal root compatible with an apical lesion in Tooth 19. At the level of Tooth 30, a coronal radiolucent zone is observed to be compatible with coronal destruction due to caries and an intraradicular radiopaque zone to be compatible with endodontic filling material (Figure 1c,d).

In the CBCT, at the level of Tooth 19, a good thickness of the interradicular septum is observed with a crown–apical height of 13.1 mm from the coronal cortex to the roof of the inferior alveolar nerve and a lingual vestibule width of 9.5 mm. On Tooth 46, a good thickness of the interradicular septum is observed with a crown–apical height of 12.9 mm from the coronal cortex to the roof of the inferior alveolar nerve and a lingual vestibule width of 10.1 mm (Figure 1e).

**Figure 2:**
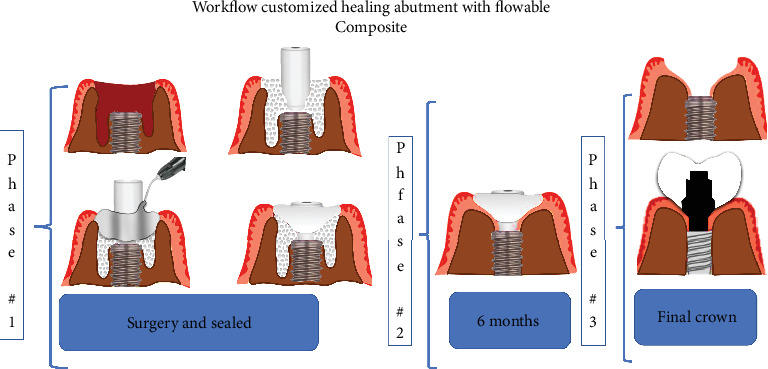
Workflow representing the step-by-step process with custom healing abutment in fluid composite in immediate implants.

Based on the literature and the description by Ruales-Carrera et al. [13], we decided to create a workflow illustration of the technique to make it easier for readers to understand (Figure 2).

**Figure 3:**
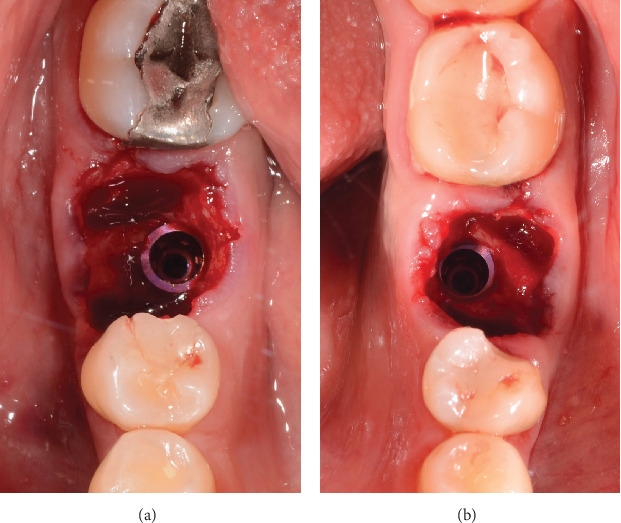
Implant protocol: (a) implant placement in Zone 19 of the MIS C1, 4.2 × 11.5 mm, and (b) implant placement in Zone 30 of the MIS C1, 4.2 × 11.5 mm.

The patient is offered the possibility of performing a single surgical procedure and thus reducing rehabilitation times. Following the technique described by Ruales-Carrera et al. [13], we performed the following steps. Minimally invasive extraction of Teeth 19 and 30 was performed, without flap lifting, and odontosection was performed at the level of the furcation with a high-speed piece and a Zekrya bur and avulsion of Teeth 19 and 30. Subsequently, the surgical zone is prepared for implants in Zones 19 and 30 at 700 RPM at the level of the bone septum at 11.5 mm depth; then the implant from the commercial company MIS C1 4.2 mm × 11.5 mm is placed in the zone of 19 and 30 (Figure 3a,b), with a primary stability of 35 NCM; and the implant was placed 1 mm subcrestal and 4 mm from the gingival margin, as reported by Gomez-Meda et al. [15].

**Figure 4:**
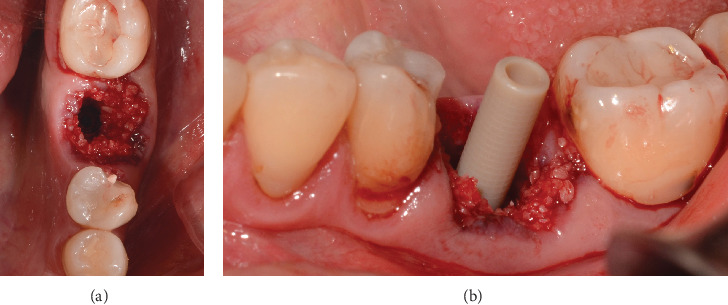
Healing abutment protocol: (a) guide bone regeneration with cortico-cancellous bone (Puros) filling the gap of the implant and the alveoli of the roots and (b) placement of the provisional abutment (PEEK).

Guided bone regeneration is performed with 0.5 g of PUROS particulate cortico allograft, with a small particle size of 250–1000 *μ*m, filling the gap between the implant and the sockets of the mesial and distal roots, and the graft is compacted (Figure 4a). This GBR is to prevent extensive tridimensional collapse of the alveolar bone, thus favoring future rehabilitation, as Covani et al. demonstrated [5]. The provisional abutment (PEEK) is placed on the implant platform, which has previously been placed with adhesive in its midcoronal portion to allow good adhesion of the flowable composite (Figure 4b).

**Figure 5:**
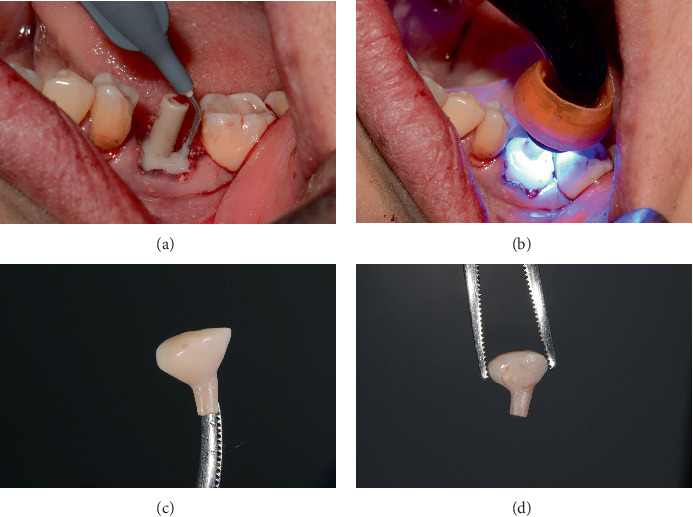
(a) Placement of the flowable composite, (b) light curing of the flowable composite, (c) polished final customized Abutment 19, and (d) polished final customized Abutment 30.

Then, flowable composite resin is placed around the peri-implant tissues, in the entire zone, to be occupied by the abutment, directly towards the bone graft. Prior to this, it is necessary to give a concave shape to the abutment of the bone graft so that when injecting the resin, we obtain an emergency profile as similar as possible to the one we planned and want to make (Figure 5a). It is then light-cured, and the abutment is extracted (Figure 5b). Excess graft attached to the abutment is removed, and we begin again to add flowable composite, creating critical and subcritical contours as González-Martín et al. reported [16]. There are three zones referring to the subgingival contour of the emergence profile of an implant restoration, and we tried to recreate this area, with a critical zone being convex to provide support to the gingival margin and a subcritical zone being straight. Subsequently, the carving of the abutment begins with an electric micromotor and buds intended for them, followed by soflex polishing discs. In later cases, the buccal and lingual profiles are not performed by more than 32°to get the final profile as Yi et al. reported [17] (Figure 5c,d).

**Figure 6:**
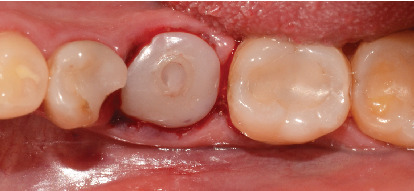
Screwed healing abutment.

The abutment should always be cleaned in saline solution. Once the excess coronal portion of the PEEK has been cut and has excellent polishing, as Ghezzi et al. reported [18], to avoid the accumulation of bacterial plaque, we take it to the mouth and screw it in using the through screw, and we seal the chimney of the implant using Teflon and subsequent flowable composite (Figure 6).

**Figure 7:**
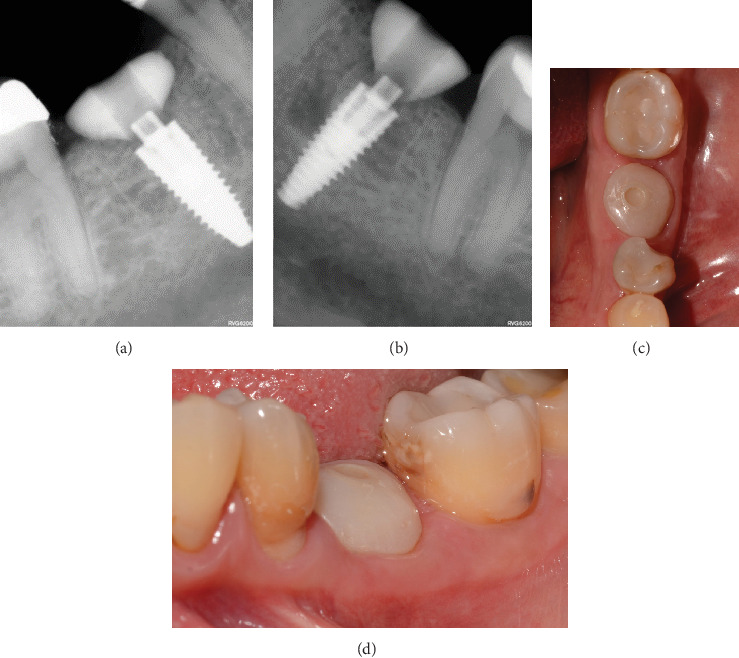
Clinical and radiographic controls: (a) postoperative periapical x-ray of Tooth 30; (b) postoperative periapical x-ray of Tooth 19; (c) clinical control 1 month after surgery, occlusal view; (d) clinical control 1 month after surgery, lateral view without any contact.

After this, sometimes a suture technique is performed with Nylon 5.0 at the level of the papillae to achieve confrontation between them, better adaptation of the soft tissue to the abutment, and prevention of any type of migration of the bone graft. Intraoperative periapical x-rays are performed, and abutment adaptations are verified (Figure 7a,b). A postoperative control was performed after 8 days, removing the sutures without any complications. Then, a control was performed 1 month after surgery where adequate healing of the peri-implant soft tissue was evident (Figure 7c,d).

**Figure 8:**
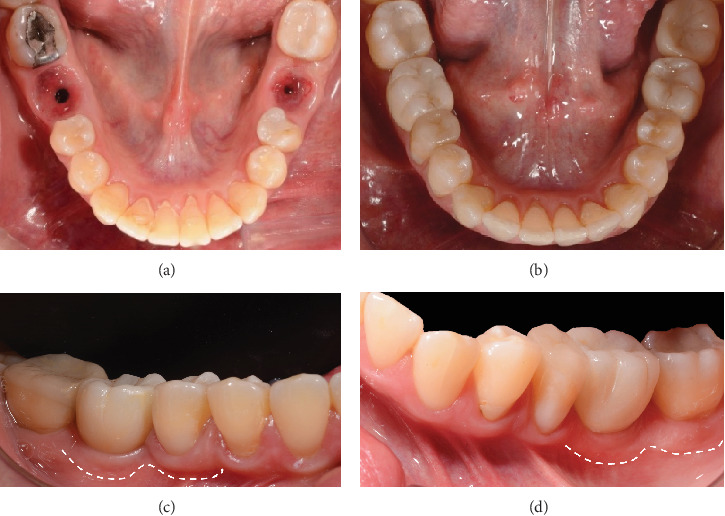
(a) Removal of customized healing abutments 4 months after surgery, (b) delivery of crowns 6 months after surgery, (c) final crown Tooth 30 maintaining the tissue and good adaptation of papillae, (d) final crown Tooth 19 maintaining tissues and good adaptation of papillae.

After 4 months, the customized healing abutments of 19 and 30 were uncovered, observing slight erythema associated with the accumulation of biofilm and the maintenance of hard and soft tissues around implants (Figure 8a). Polishing of the abutments was again carried out in internal areas, and they were adapted and screwed. Weeks later, the definitive restoration process began with full-contour monolithic crowns in cement-retained zirconia screwed to titanium bases in Zones 19 and 30, which were cement-retained 6 months after the placement of the implants, and we can see the maintenance tissues and good adaptation of papillae (Figures 8b, 8c, and 8d). This tested restorative system was highly effective and reliable for restoring occlusal function, showing 100% survival and success rates in a 6-year prospective clinical study by Sorrentino et al. [19], which makes it a good option to rehabilitate this clinical case.

**Figure 9:**
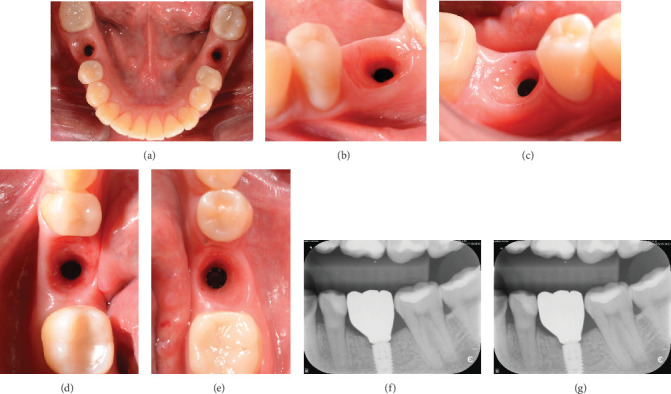
(a) Control 1 month after crown delivery; (b) lateral view, peri-implant mucosa of Implant 19; (c) lateral view, peri-implant mucosa of Implant 30; (d) occlusal view, peri-implant mucosa of Implant 19; (e) occlusal view, peri-implant mucosa of Implant 30; (f) periapical x-ray of Zone 19, 1 month after crown screwing; (g) periapical x-ray of Zone 30, 1 month after crown screwing.

One month after screwing the crowns, postsurgical control is carried out, the 19 and 30 crowns are removed to evaluate the peri-implant soft tissue, where adequate healing is evident, without signs of inflammation (Figures 9a, 9b, 9c, 9d, and 9e), and final periapical x-rays are taken (Figure 9f,g).

## 3. Discussion

In search of a safe procedure, with reduced time and affordable cost for patients, we decided to document two clinical cases and carry out a narrative review on customized healing abutments in flowable composite in immediate postextraction implants. Customized healing abutments on immediate implants have the function of maintaining the volume of keratinized mucosa and mucogingival line, creating an emergence profile for the future prosthesis and reducing the risk of peri-implantitis. The development of healthy peri-implant soft tissues is essential to achieve the aesthetics and biological success of implant-supported restorations in all stages of healing and tissue maturation, starting with provisionalization, which can be performed in some cases, from the same day of the placement of the dental implants, through noncontact loads and occlusion such as personalized abutments on dental implants [20]. Successful healing of peri-implant soft tissues is extremely important to maintain the health of dental implants, because it allows a biological seal that protects the bone tissue [1].

From the biomechanical point of view, it is expected that a customized abutment will enhance the process of osseointegration. This is because the transfer of load creates a specific alteration in the biomechanical environment in the area surrounding the implant, which sends mechanical signals that prompt the deposition of bone on the porous surface [21].

The abutment taper configuration had a slight effect on bone remodeling but exerted a significant effect on the peri-implant gingiva above the implant platform via hydrostatic pressure [22].

Knowing this about the principles, our clinical case meets the need to maintain hard and soft tissues with the creation of an immediate abutment after tooth extraction with the ideal biomechanical characteristics, to avoid future complications. In this study, we perform an evaluation and monitoring of the implant tissues with clinical evaluation and periapical x-rays, to assess the quantity and quality of the surrounding bone, as well as its osseointegration. We verify the adaptation of the abutment, and once placed, we verify that there are no signs of occlusal overload, leaving it out of contact. The management of surfaces influences the long-term maintenance of dental implants. A correct choice of the headmasters in terms of materials of the hand tools and implants surfaces is fundamental to solve implant-prosthetic problems without creating iatrogenic damage and future peri-implantitis. It is highly recommendable to perform the implant maintenance protocol of oral hygiene in both domiciliary and professional ways. In case we find tissue diseases around the implant once it has been restored, we have several treatment options; in accordance with Amodeo et al. [23], nonsurgical therapy alone can resolve peri-implant mucositis, but not peri-implantitis.

Also, the soft tissue architecture surrounding the extracted tooth must be maintained during extraction and placement of the dental implant. The shape of these tissues most observed after an atraumatic extraction is oval, triangular, square, and trapezoidal, depending on the tooth to be treated [24]. The main objective of the provisional is to imitate the natural profile of an emerging tooth that will receive the future implant prosthesis. The shape of the provisional restoration is an important factor in maintaining stable gingival contours. However, the loss of references in the horizontal and vertical plane due to the collapse of soft or hard tissue after tooth extraction can present a clinical challenge, which is why it is important to perform a minimally invasive extraction, avoiding flap lifting, incisions, and osteotomies, since they may affect the peri-implant hard and soft tissue levels later [12, 13]. One way to properly direct the contour of the soft tissues is to reproduce the shape of the root in the cervical third of the newly extracted tooth and follow one of the contouring concepts described previously depending on the area [24]. In the present study, in both cases, a minimally invasive extraction was performed, without incisions, flap lifting, osteotomies, and performing odontosection at the furcation level to minimize the risk of fracture of the buccal bone.

In this investigation, both cases were managed with PEEK abutments and flowable composite. PEEK has been proposed as a good material to be used in oral implantology due to its excellent biological properties, which are associated with good mechanical stability. This material has been widely used, and biomechanical studies have indicated that PEEK has an elastic modulus similar to that of bone tissue and a high tensile strength, which helps to counteract the occlusal forces generated during chewing [25]. These biomechanical characteristics have been confirmed by case reports that showed excellent results related to the use of PEEK as a material for implant-supported prostheses [26]. In the present study, in both cases, PEEK and flowable composite were used to make the customized healing abutments. It was observed that 30 days after placement of the personalized abutment, the peri-implant tissue presented a good clinical condition, with no evidence of inflammation and adequate healing. The correct healing of the peri-implant soft tissue related to the use of PEEK may be associated with the good biological properties of said material that have been reported in several studies. An in vitro study demonstrated that PEEK induces the adhesion, proliferation, and viability of fibroblasts due to the overexpression of proteins that increase contractility and cell adhesion (integrins) and a better synthesis of proteins from the connective tissue matrix (Type I collagen and fibronectin) [27]. Other in vitro studies have indicated that PEEK impairs the adhesion of important biofilm-forming microorganisms, such as *Streptococcus mutans* and periodontopathogens such as *Porphyromonas gingivalis* and *Fusobacterium nucleatum* [28]. However, a disadvantage of PEEK is considered to be its inert surface that leads to inadequate bonding with dental materials [29].

In the management of the two cases presented, flowable composite was chosen as a complementary material to PEEK. The composite has a low elastic modulus but high fracture resistance and tensile strength. Both ceramic and composite abutments have a similar failure rate during accelerated surgery in vitro, with fatigue testing, suggesting that composite could be used to fabricate healing abutments [1]. Menchini-Fabris et al. [14], in their retrospective study with a 1-year follow-up, demonstrated that a protocol consisting of the placement of an implant with immediate loading in postextraction sockets and the creation of a customized provisional healing abutment made of PEEK + composite could represent a valid treatment strategy for the rehabilitation of nonrehabilitable teeth.

While it is true that the literature supports the use of polymeric materials such as composite and flowable composite indicated to capture the contour of the postextraction tooth in the immediate placement of the implant [1], the physical and mechanical properties of the composites depend on the type of imaging lamp, photopolymerization, thickness of material layers, and curing technique [18, 30]. These factors guarantee a higher percentage of polymer conversion, reducing leaching, degradation, and release of residual monomers, which have been associated with cytotoxic effects on epithelial cells and fibroblasts that have been shown to have negative effects on cell viability, directly affecting soft tissue healing around customized healing abutment [31].

Additionally, the healing and adhesion of the tissue to the pillar are directly related to a finishing, polishing, and shine protocol, reducing the accumulation of bacterial plaque, minimizing irritation and erythema, and contributing to tissue stability [30]. Studies have shown that gingival fibroblasts show a greater ability to adhere to abutments with polished surfaces [1, 31]. For this, the use of abrasive discs or composite polishing rubbers requires strictly following the sequence recommended by the manufacturer, and it is essential to accompany this process with abundant irrigation to prevent overheating and structural damage to the material. As an additional benefit, this method helps to increase the contact angle and hydrophilicity of the resin, thus improving tissue adhesion and healing processes [18].

Several reports have suggested workflows for creating temporary implant-supported restorations. Bichacho and Landsberg recommend [32] the use of a cervical contouring concept using a custom temporary restoration design to reshape the soft tissue around the implants with a primary focus on the marginal soft tissue levels and zenith position. Rompen et al. defended [33] the use of a concave transmucosal profile to minimize vestibular gingival recession. Recently, Su et al. defined [24] two different areas within the transgingival zone, based on the response of the peri-implant gingival tissues to the abutment and modifications of the crown contour and the critical and subcritical contours. The critical contour is the most superficial area of the restoration and will influence the gingival level and location of the zenith, while the subcritical contour corresponds to the deepest area of the restoration, which influences the support of the peri-implant soft tissue and, consequently, the gingival color [16].

Unlike prefabricated abutments such as healings or healing screws, these are not capable of adequately maintaining the necessary profiles for a future restoration on implants, which is why this would be the answer to why it is better to manufacture completely customized healing abutments. To avoid future peri-implant bone loss, customized healing abutments must be manufactured in a correct shape and manner [16]. Modifications to the restorative emergence profile at the critical and subcritical contours are essential to optimize the peri-implant soft tissue architecture. In the case of immediate implants, the critical contour must support the architecture of the gingival margin, while the subcritical contour can be designed to provide regenerative space through a concave configuration. These contours are dynamic areas that can be modified during the conditioning of mature tissues in late cases.

Although the critical contour affects the gingival margin and level position, changing the convexity of the subcritical contour can optimize the soft tissue profile [16]. Yi et al., in their study, demonstrated [17] that overcontoured implant-supported prostheses are associated with peri-implantitis. The emergence angle or profile ≥ 30°, a convex emergence profile, and a splinted midposition were identified as factors associated with an increased risk of marginal bone loss and peri-implantitis. In the present study, with respect to the shape of the customized abutment in the cervical area (critical contour), it was made in an oval shape, following the anatomy of the postextraction socket, maintaining the original contour of the tooth on the palatal and interproximal surfaces and trimming between 0.5 and 1 mm on the vestibular surface. Regarding the shape of the subcritical contour, it was made as concave as possible and with an angulation of less than 30°.

To achieve the desired tissue stability, González-Martín et al. indicate [16] that the provisional restoration must be formed in accordance with the following guidelines:

✓ A critical contour that supports the existing gingival margin and papillae height. The original contour of the tooth is maintained palatally and interproximally, while buccally it could be trimmed by 0.5–1 mm to favor a slight coronal displacement of the gingival margin after the healing process.
✓ A subcritical contour as concave as possible to allow space for the clot and graft material to stabilize and potentially reconstruct the bone crest.
✓ A smooth, polished surface will help create a smooth transition and minimize contamination during healing. The selection of the dimensions of the temporary is key to obtaining an optimal result. Balancing the need for space for peri-implant connective tissue and the space to create a smooth subcritical contour profile is not always easy; it depends on the depth of the implant, the bucco-lingual position, and the height of the platform, and all of these details should be carefully evaluated due to their influence on the potential design and configuration of the prosthesis [16].

Yu et al. indicate [34] that using particulate bone graft results in the formation of vital bone and soft tissue 4–6 months after the procedure, which agrees with the definitive rehabilitation stage in immediate implants. They show that in general there is a 13% reabsorption of the material and that the most important thing is a material that serves as a barrier to keep the clot in position that will help with the formation of bone tissue throughout the healing period [34].

Small and Tarnow showed that the buccal gingival margin stabilizes 3 months after abutment connection. It is recommended that any subcritical facial contour alteration be performed once the gingival margin is stable [35].

Considering that we can use prefabricated or customized abutments, Chokaree et al. in their study focused on three major differences between prefabricated and customized healing abutments, including abutment dimensions, the abutment emergence profile, and the emergence angle. Customized healing abutments are made to the size of the socket and are concave at their emergence, reaching 20 or 30°, while prefabricated abutments are small and straight, reaching a maximum of 12 or 15°, which is sometimes insufficient for a final restoration [36].

Ruhstorfer et al. [37] indicated that the use of customized healing abutments may reduce the patient chair time during prosthesis insertion while still achieving an aesthetically pleasing result. With individually shaped peri-implant soft tissue, the need for stepwise displacement and compression of the mucosa is minimized or eliminated when the final restoration is placed and also can be used in immediate or late implants.

Among the factors evaluated in our patient was the amount of keratinized tissue, resulting in a biological width of 4 mm. The contours of the hard and soft tissues were maintained after extraction and implant placement with the custom abutment. No papillae recession was observed, and second surgeries were avoided, which would not have been possible with the use of conventional abutments.

Perez et al. [38] demonstrated that a customized healing abutment group showed the most favorable outcomes in case of immediate implant that received a peri-implant bone grafting procedure versus standard healing abutment group.

Our limitation in this article is the sample size, so it would be important in future research to use representative sample sizes to strengthen the evidence base for the use of custom abutments in dental implants.

## 4. Conclusion

In this study, it was demonstrated that the use of the customized healing abutment with PEEK and flowable composite immediately after implant placement following tooth extraction maintains the volume of the soft and hard tissues around the implants at the time of definitive prosthesis.

There must be interdisciplinary management in all cases, with assertive communication between the surgeon and the prosthetist, since the objective is to perform a single procedure where both specialties act at a single time, and although the technique is faster, atraumatic, and economical, it must be created and respect the emergency profile zones in the abutment, and a strict finishing, polishing, and gloss protocol must be developed for it to be successful.

## Data Availability

The data that support the findings of this study are available from the corresponding author upon request.
